# One (effect) size does not fit at all: Interpreting clinical significance and effect sizes in depression treatment trials

**DOI:** 10.1177/0269881120922950

**Published:** 2020-05-25

**Authors:** Fredrik Hieronymus, Sameer Jauhar, Søren Dinesen Østergaard, Allan H Young

**Affiliations:** 1 Department of Clinical Medicine, Aarhus University, Aarhus N, Denmark; 2 Department of Affective Disorders, Aarhus University Hospital, Aarhus N, Denmark; 3 Department of Psychological Medicine, Institute of Psychiatry, Psychology and Neuroscience, King’s College London, London, United Kingdom; 4 Department of Psychological Medicine, Centre for Affective Disorders, Institute of Psychiatry, Psychology and Neuroscience, King’s College London, London, United Kingdom; 5 South London and Maudsley NHS Foundation Trust, London, United Kingdom

**Keywords:** Depression, antidepressants, meta-analysis, efficacy, clinical trials

## Abstract

The efficacy of antidepressants in major depressive disorder has been continually questioned, mainly on the basis of studies using the sum-score of the Hamilton Depression Rating Scale as a primary outcome parameter. On this measure antidepressants show a standardised mean difference of around 0.3, which some authors suggested is below the cut-off for clinical significance. Prompted by a recent review that, using this argument, concluded antidepressants should not be used for adults with major depressive disorder, we (a) review the evidence in support of the cut-off for clinical significance espoused in that article (a Hamilton Depression Rating Scale standardised mean difference of 0.875); (b) discuss the limitations of average Hamilton Depression Rating Scale sum-score differences between groups as measure of clinical significance; (c) explore alternative measures of clinical importance; and (d) suggest future directions to help overcome disagreements on how to define clinical significance. We conclude that (a) the proposed Hamilton Depression Rating Scale cut-off of 0.875 has no scientific basis and is likely misleading; (b) there is no agreed upon way of delineating clinically significant from clinically insignificant; (c) evidence suggests the Hamilton Depression Rating Scale sum-score underestimates antidepressant efficacy; and (d) future clinical trials should consider including measures directly reflective of functioning and wellbeing, in addition to measures focused on depression psychopathology.

## Introduction

In a recent narrative review on the risks and benefits of antidepressants, Jakobsen, Gluud and Kirsch conclude that ‘antidepressants should not be used for adults with major depressive disorder’ (Jakobsen et al., 2019). They arrive at this recommendation by discarding the criterion for clinical significance in depression once endorsed by the National Institute for Health and Care Excellence – a three-point change on the 17-item Hamilton Depression Rating Scale (HDRS) – as being ‘presumably too small’ (Jakobsen et al., 2019). Instead they suggest that a seven-point HDRS difference, or a standardised mean difference (SMD) of 0.875, should be the cut-off for a ‘*minimally important difference*’ (MID). An SMD of 0.875 means the average person receiving antidepressants has an endpoint score being 0.875 standard deviations lower than that of the average person given placebo, which, given the variability observed in antidepressant treatment trials, translates to roughly seven HDRS points.

Although antidepressants, which have an SMD of roughly 0.3 compared with placebo (Hieronymus et al., 2016a; Jakobsen et al., 2017; Kirsch et al., 2008; Turner et al., 2008), are far from meeting this 0.875 SMD requirement, the choice of cut-off is perplexing. Not only is 0.875 considerably higher than effect sizes for most treatments used in psychiatry and general medicine (Leucht et al., 2012), it also implies that for antidepressants to be considered *minimally* efficacious, almost all patients treated with an antidepressant need to achieve remission (see below). Arguments on the cut-off for clinical relevance in depression are not new (Turner and Rosenthal, 2008) and, given the continuing interest, it is important to understand the provenance of the proposed seven-point HDRS cut-off as well as of the psychometric properties of the HDRS in general. The aim of this paper is therefore to provide a balanced perspective on interpretation and utility of the scale to prevent potentially misleading narratives.

### The validity of the seven-point HDRS cut-off

In 2015, Moncrieff and Kirsch published a short communication (Moncrieff and Kirsch, 2015), utilising data from large linkage analyses of major depressive disorder (MDD) patients treated with mirtazapine (Leucht et al., 2013). The Leucht analysis linked data from 43 mirtazapine trials (obtained from Organon) in people with MDD, utilising the Hamilton Depression Rating Scale 17-item version (HAMD-17) and Clinical Global Impression Severity (CGI-S) and Improvement (CGI-I) scales. This was then presented in graphical form to enable interpretation of the relationship between changes in HAMD, CGI-I and CGI-S. Moncrieff and Kirsch noted the mean HDRS rated improvement in patients meeting the CGI-I category of ‘*minimally improved*’ was seven points. Thus, if a depressed patient entered a trial with a score of, for example, 25 points on the HDRS and ended the trial with a score of 18 points, that patient is likely to have received a CGI-I rating of ‘*minimally improved*’.

There are reasons to doubt the validity of this seven-point cut-off signifying ‘*minimal improvement*’ in an individual patient. First, the authors of the original analysis did not suggest the seven-point cut-off, rather they commented that ‘[a] CGI-I score of 3 (“minimally improved”) corresponds to a reduction from baseline in the total HAMD-17 score of between 25% and 35%’. By choosing a relative cut-off, the authors recognised those with less severe illness require a smaller absolute decrease in HDRS scores for a clinically significant difference. The lower estimate, 25%, means the seven-point cut off would be appropriate for people with a baseline score of 28, that is, far higher than the average people participating in antidepressant treatment trials (Kirsch et al., 2008). Second, most depression treatment trials enforce a minimum HDRS score as inclusion criterion and this is usually known to the HDRS rater. Because there is often pressure to recruit patients, this practice can lead to inflated baseline scores (Kobak et al., 2010; Mundt et al., 2007). If HDRS baseline scores are inflated, then all subsequent HDRS change scores are as well and, consequently, HDRS change scores corresponding to specific CGI-I categories, such as the ‘*minimally improved*’ category, will also be inflated. Third, although knowing the HDRS and CGI correlate is interesting, a ‘minimal improvement’ in CGI-I is still an approximation, for which we do not necessarily know the meaning, for example, in terms of functioning.

These reservations notwithstanding, Moncrieff and Kirsch took this cut-off one step further, suggesting it should not only be used to signify a minimal improvement as compared with baseline for an individual patient, but also as cut-off for the minimally important difference (MID) between treatment groups. This transformation is problematic because endpoint scores consist of a mixture of patients. Some will show only ‘minimal improvement’, others will not improve at all (who may well have dropped out of treatment) and others will have improved markedly. Because patients vary greatly in treatment outcomes, assessing whether antidepressants have clinically significant benefits over placebo necessarily entails assessing how patients distribute across these categories, for example, if there is a larger proportion of cases who are no longer depressed in the active treatment group (Dworkin, 2016). Moncrieff and Kirsch offer no rationale for their unintuitive transformation, other than the self-evident observation that within-patient and between-group differences are measured by the same units (i.e. HDRS points): ‘[equipercentile] linking has been used to establish the clinical relevance of pre–post treatment differences. We propose that it can also serve as an empirically validated method of evaluating the clinical significance of drug-placebo differences, since these are also frequently calibrated in terms of differences on the Hamilton scale’ (Moncrieff and Kirsch, 2015).

Arithmetically, it is also questionable whether it is theoretically possible to attain a drug-placebo difference of the magnitude that Jakobsen, Gluud and Kirsch have mandated (Jakobsen et al., 2019). As illustrated, for example, in a 2017 meta-analysis (Jakobsen et al., 2017) most placebo groups have endpoint scores below 14 HDRS points. Taking ⩽7 HDRS points as a cut-off for remission, the MID championed above implies almost all patients treated with antidepressants need to attain remission as assessed by the HDRS. Because healthy volunteers average about three HDRS points (Zimmerman et al., 2004), there is very little room for dropouts and/or residual symptoms and/or treatment non-responders due to, for example, misdiagnosis or presence of individuals with treatment-resistant depression. Given the average length of most antidepressant trials (usually 6 or 8 weeks), a goal of almost 100% remission might therefore be too high a bar to set for a *minimal* improvement over placebo.

### The validity of the HDRS sum score

The HDRS has been considered the gold standard depression rating instrument for decades and the majority of antidepressant treatment trials have used it as primary outcome measure (Bagby et al., 2004). Any effort at evaluating the efficacy of, for example selective serotonin reuptake inhibitors (SSRIs) or serotonin-noradrenaline reuptake inhibitors (SNRIs), is thus heavily influenced by the psychometric properties of the HDRS. Because depression is a highly heterogeneous illness, many symptoms measured by the HDRS may reflect factors other than depression symptoms (e.g. age or somatic comorbidities) and thus be expected to persist even if depression remits. Likewise, not all symptoms included in the HDRS are present in all patients at baseline but may still vary over time, which might also increase variance. Further, the HDRS includes items measuring gastrointestinal and sexual dysfunction, which are common antidepressant side effects and may therefore be expected to worsen with antidepressant treatment (Bech, 2010). Conversely, the HDRS also includes three items measuring insomnia, thus making it theoretically possible that a sedative drug with no beneficial effect on, for example, mood or anhedonia would separate from placebo with respect to HDRS sum score (Moncrieff, 2007). These factors may partly explain the observed disconnect between HDRS- and patient-rated remission (Zimmerman et al., 2012).

One early attempt to improve measuring of depression severity was undertaken by Per Bech (Bech et al., 1975). Bech extracted a unidimensional six-item subscale from the 17 items included in the original HDRS. This subscale, developed well before introduction of modern antidepressants, has several decades later been shown to yield 20–30% larger drug-placebo separation than the full HDRS scale (Faries et al., 2000; Hieronymus et al., 2016a). Its constituent items, that is, depressed mood, feelings of guilt, work and interests, psychomotor retardation, psychic anxiety and general somatic symptoms – which measures fatigability and loss of energy – correspond well to symptoms that explain most variance in patient-assessed impairment of functioning (Fried and Nesse, 2014). This suggests these are the symptoms that matter most to patients. Add suicidal ideation to this list and one has the collection of symptoms where serotonergic antidepressants most clearly, and rapidly, separate from both placebo (Hieronymus et al., 2016a, 2016b, 2019; Lisinski et al., 2019; Naslund et al., 2018) and psychotherapy (Boschloo et al., 2019). By contrast, serotonergic antidepressants do *not* excel on HDRS items such as insomnia, agitation, somatic anxiety, gastrointestinal symptoms, sexual dysfunction and weight loss (Table 1); especially not in people with comparatively mild depression (Hieronymus et al., 2019). Taken together, the mean HDRS change gives an incomplete and noisy picture. In fact, the effects of antidepressants are not small and non-specific, as suggested (Moncrieff, 2007), rather they are sizeable and affect preferentially those symptoms that depressed persons appear to judge most relevant (Fried and Nesse, 2014; Hieronymus et al., 2016a, 2016b, 2019; Lisinski et al., 2019; Naslund et al., 2018).

**Table 1.** Effect sizes for various HDRS-derived outcome parameters.

Outcome measure	Standardised mean difference
HDRS-17-sum	0.27
HDRS-6 subscale	0.35
HDRS item 1: Depressed mood	0.40
HDRS item 2: Feelings of guilt	0.26
HDRS item 3: Suicidality	0.22
HDRS item 4: Insomnia, early	0.08
HDRS item 5: Insomnia, middle	0.07
HDRS item 6: Insomnia, late	0.13
HDRS item 7: Work and activities	0.23
HDRS item 8: Psychomotor retardation	0.21
HDRS item 9: Psychomotor agitation	0.08
HDRS item 10: Psychic anxiety	0.30
HDRS item 11: Somatic anxiety	0.06
HDRS item 12: Somatic symptoms, gastrointestinal	-0.02
HDRS item 13: Somatic symptoms, general	0.16
HDRS item 14: Genital symptoms	-0.01
HDRS item 15: Hypochondriasis	0.12
HDRS item 16: Loss of weight	-0.06
HDRS item 17: Lack of insight	0.07

Reproduced from Hieronymus et al. (2016a). The effect size estimates are from a pooled patient-level analysis of data from 6669 adults treated with either an SSRI or a placebo in short-term MDD trials. The HDRS-6 subscale includes HDRS items 1, 2, 7, 8, 10 and 13.

HDRS: Hamilton Depression Rating Scale; MDD: major depressive disorder; SSRI: Selective serotonin reuptake inhibitor

An alternative way of looking at depression outcome data is to transform scale scores into clinically relevant dichotomous metrics, such as response (⩾50% decrease as compared with baseline) and remission (HDRS endpoint score ⩽7). It has been argued that such transformations are inappropriate, partly because they can inflate minute differences between treatments, depending on how endpoint scores distribute around the cut-off point, but also because they give no information on possible deleterious effects; for example, if significant worsening is more common on one treatment than another) (Jakobsen et al., 2019). However, in the case of depression, the drug-placebo differences in response and remission are of a comparable absolute magnitude (Hieronymus et al., 2016b). This likely indicates that both are primarily driven by more patients below the lower of the two cut-offs (remitters) in the drug group and a corresponding accumulation of patients above the higher cut-off (non-responders) in the placebo group. This, in conjunction with the fact that significant worsening is exceedingly uncommon in depression trials and that non-response is more common on placebo than on pharmacotherapy (Vittengl et al., 2016), suggests these theoretical concerns have little relevance here.

Given the considerable heterogeneity of the depressive phenotype, there may be individual differences in response to antidepressants. And if, as suggested by the significant efficacy seen in relapse prevention studies (Geddes et al., 2003; Young, 2001), it is the case that some people respond very well to a particular antidepressant (i.e. remitters), whereas others derive little to no benefit (i.e. those classified with treatment-resistant depression), then dichotomous outcome measures may better reflect clinical reality than average HDRS differences, because the latter metric implies that all treated patients will have the same effect of treatment. Such a non-constant effect is compatible with symptom-level differences in efficacy, as described above, because some symptoms (e.g. depressed mood and psychic anxiety) are present to a large degree in almost all patients and may thus also improve in almost all patients, whereas other symptoms are much more likely to be absent at baseline and thus to have no room to improve (Hieronymus et al., 2019). Nevertheless, concerns regarding the psychometric properties of the HDRS sum score remain, and it may well be that response and remission rates are underestimated due to, for example, the HDRS capturing common antidepressant side effects (Østergaard, 2018).

### How do we move forward?

If the criterion for clinical significance detailed above (Jakobsen et al., 2019) is too strict, what does constitute a reasonable cut-off? There is unfortunately no clear answer to this question, as no one has yet figured out how to reduce the mix of patient trajectories (dropouts, partial responders, non-responders, remitters, etc.) and dose-dependent symptom-level effects into one incontrovertible cut-off. And if, as seems likely, antidepressants do not have the same effect in all patients, then the idea of a cut-off is itself misguided. The issue then becomes how to best identify patients for whom treatment is, on balance, beneficial. Relatedly, it should be acknowledged that demonstrating efficacy is not limited to observations from acute-phase trials. There is significant evidence to support that antidepressants prevent recurrent episodes of depression in patients who have responded to treatment (Geddes et al., 2003; Young, 2001).

We thus agree with the sentiments of Jakobsen et al. who in 2014 concluded ‘when surrogate outcomes or continuous outcomes are used to assess intervention effects, it is often unclear if a given statistical significant effect has any patient relevant clinical significance’ and suggested that ‘clinical researchers in close cooperation with patients and relatives must somehow consent on the quantification of the “minimal relevant clinical differences” as well as the relevant outcomes to be assessed’ (Jakobsen et al., 2014). We would espouse this nuanced view, instead of a simplistic analysis in which the ‘to be or not to be’ of antidepressants is contingent on which misleading and arbitrary cut-off is chosen, without interpretation of the outcome measure itself.

Other rating instruments may more accurately measure disease-specific psychopathology than the full HDRS-17 – for example, the Montgomery-Åsberg Depression Rating Scale (MADRS) or the HDRS-6 (Bech et al., 1975; Montgomery and Asberg, 1979) – although the MADRS also suffers from some of the problems identified above with the full HDRS-17. This was shown in an analysis of the full MADRS, HDRS-17 and their ‘melancholia’ sub-scales (MADRS-5 and HDRS-6), revealing only the HDRS-6 to demonstrate unidimensionality (Bech et al., 2014).

We also suggest future treatment studies should consider routinely including measures of subjective wellbeing and functioning (Bech, 2018). In this context, it is notable that the clearest indications of antidepressant efficacy in the recent PANDA trial that included people with depression for which there was clinical uncertainty as to the value of adding antidepressant treatment came not from the self-report depression rating scales (Patient Health Questionnaire, PHQ-9 and Beck Depression Inventory, BDI-II) but from measures of anxiety and overall mental health-related quality of life (Generalized Anxiety Disorder 7-item scale, GAD-7, Short Form 12-item Mental Health Survey, SF-12 Mental Health; Lewis et al., 2019).

Until more data are available from trials reliably capturing change in functioning and wellbeing, the simple option of looking at response and/or remission rates may be a more informative and accessible way of informing outcomes than use of a rating scale in isolation. It does seem likely that most clinicians, patients and relatives would agree that a person who demonstrates almost no symptomatology is better off than one who displays a considerable amount of symptomatology, which is probably what the response and remission differences reflect.

In summary, although there is no doubt a need to delineate drugs that are, on balance, safe and effective from those that are not, the idiosyncratic way in which some authors rely on highly questionable figures – such as a cut-off for clinical significance that is theoretically misguided and in practice equates *minimal* improvement with near 100% remission – illustrates a deeper issue: criticism against antidepressants is so commonplace that critics need no longer provide sound evidence-based arguments (Jauhar and Young, 2018). There is a need for the field, including researchers, journal editors, peer reviewers and policy makers, to scrutinise misinterpretations in spite of, or perhaps because of, what may appear to be captivating and attention-grabbing headlines. We suggest average HDRS sum-score differences from short-term trials are inadequate as a sole measure of clinical significance of antidepressants, that analyses relying solely or primarily on these will likely underestimate antidepressant efficacy and that future evidence syntheses would benefit from a degree of nuance. As a pertinent example, the statement that ‘antidepressants should not be used for adults with major depressive disorder’ (Jakobsen et al., 2019) is – at best – unfounded.
